# Prediction of persistent acute kidney injury in postoperative intensive care unit patients using integrated machine learning: a retrospective cohort study

**DOI:** 10.1038/s41598-022-21428-5

**Published:** 2022-10-12

**Authors:** Xuandong Jiang, Yongxia Hu, Shan Guo, Chaojian Du, Xuping Cheng

**Affiliations:** Intensive Care Unit, Affiliated Dongyang Hospital of Wenzhou Medical University, No. 60 Wuning West Road, Jinhua, Dongyang, Zhejiang People’s Republic of China

**Keywords:** Kidney, Kidney diseases, Nephrology, Risk factors, Disease prevention, Prognosis

## Abstract

Acute kidney injury (AKI) often occurs in patients in the intensive care unit (ICU). AKI duration is closely related to the prognosis of critically ill patients. Identifying the disease course length in AKI is critical for developing effective individualised treatment. To predict persistent AKI at an early stage based on a machine learning algorithm and integrated models. Overall, 955 patients admitted to the ICU after surgery complicated by AKI were retrospectively evaluated. The occurrence of persistent AKI was predicted using three machine learning methods: a support vector machine (SVM), decision tree, and extreme gradient boosting and with an integrated model. External validation was also performed. The incidence of persistent AKI was 39.4–45.1%. In the internal validation, SVM exhibited the highest area under the receiver operating characteristic curve (AUC) value, followed by the integrated model. In the external validation, the AUC values of the SVM and integrated models were 0.69 and 0.68, respectively, and the model calibration chart revealed that all models had good performance. Critically ill patients with AKI after surgery had high incidence of persistent AKI. Our machine learning model could effectively predict the occurrence of persistent AKI at an early stage.

## Introduction

Acute kidney injury (AKI) refers to the sudden loss of excretory renal function and is a common and complex clinical syndrome that occurs in patients in the intensive care unit (ICU)^1^. The clinical manifestations vary based on the duration and severity, and the required methods of treatment also differ. The duration of AKI is an important factor in predicting the mortality of critically ill patients^2^. A systematic review reported that AKI duration was independently correlated with patient mortality and the occurrence of cardiovascular events^3^. In 2017, the Acute Dialysis Quality Initiative (ADQI) Group classified AKI into transient and persistent AKI based on whether the AKI persists for more than 48 h. Predicting the course of AKI can help identify phenotypes that require different treatment regimens, thereby facilitating individualised management. Guidelines recommend that when persistent AKI is diagnosed, clinicians should carefully reassess the patient, re-evaluate the aetiology of AKI, and re-examine the treatment regimen^4^. For patients with transient AKI, unnecessary examinations and treatments, such as renal replacement therapy (RRT), can be avoided, as their renal function will recover in a short time^5^. Therefore, the duration of AKI holds clinical significance.

However, early identification of persistent AKI is difficult, and traditional urine biochemistry^6^ and the renal resistive index^7^ exhibit poor prediction results. The latest research revealed that prediction models based on big data from modern electronic case systems combined with machine learning algorithms were more accurate than traditional methods^8^. In particular, the latest integrated machine learning algorithms combine the best-performing algorithms to create an integrated model to achieve a better modelling effect, which has begun to make important contributions in various fields, such as industry, agriculture, education, and medicine^9–12^.

Therefore, this study aimed to predict the occurrence of persistent AKI in critically ill surgical patients, based on machine learning algorithms and integrated models, for accurate early identification of high-risk patients.

## Methods

### Study design and research participants

Our study was reported according to the Transparent Reporting of a multivariable prediction model for Individual Prognosis Or Diagnosis (TRIPOD) statement^13^. A retrospective study was conducted including 955 patients with AKI who were first admitted to the ICU of Dongyang People’s Hospital between January 1, 2010, and September 30, 2020. External validation was performed using the MIMIC III dataset^14^, a freely accessible online critical care database. The inclusion criteria were patients who were first admitted to the ICU and underwent surgical treatment. The exclusion criteria were chronic kidney disease diagnosis, age < 18 years, ICU stay < 48 h, AKI diagnosis before ICU admission, or missing values of variables > 20%.

This study followed all related local guidelines and regulations, including the human genetic-related research regulations. This study was approved by the Ethics Committee of Dongyang People’s Hospital (Dong Ren Yi 2022-YX-056). The need for obtaining informed consent was waived by the local Ethics Committee (Ethical Committee of Dongyang People’s Hospital) because of the retrospective and observational study design. The data were anonymised before analysis. One author (X.J.) obtained permission to access the Medical Information Mart for Intensive Care (MIMIC) database after the completion of “Protecting Human Research Participants,” an online training course launched by the National Institutes of Health (Certification Number: 7632299).

### Data collection

We collected data using the medical record information mining software provided by Shanghai Le9 Healthcare Technology Co., Ltd. The retrieved data included the following: (1) basic clinicodemographic information (age, sex, disease severity [Acute Physiology and Chronic Health Evaluation (APACHE) II score, Sepsis-related Organ Failure Assessment (SOFA) score], duration of operation, RRT, urine volume, and complications); (2) AKI-related drugs such as antibiotics, diuretics, and contrast agents; (3) laboratory measurements on the first day of ICU admission; and (4) vital signs on the first day of ICU admission, including maxima, minima, and averages.

### Diagnostic criteria

AKI was defined and staged according to the 2012 Kidney Disease: Improving Global Outcomes criteria^15^ as follows: increase in serum creatinine (Scr) by 50%, increase in Scr by 26.5 mol/L within 2 days, or urine volume < 0.5 mL/kg/h for 6 h. Baseline Scr was defined as the lowest Scr level within 6 months before ICU admission^16^. For patients without previous Scr data, it was estimated using the following formula^17^: Scr = 0.74–0.2 (if female) + 0.08 (if black) + 0.0039* age (in years). Based on the 2017 expert consensus^4^, persistent AKI was defined as a duration of AKI > 48 h; otherwise, it was designated as transient AKI.

The primary endpoint was the incidence of persistent AKI. The secondary outcomes included hospitalisation mortality, length of ICU stay, length of hospital stay, mechanical ventilation duration, and hospitalisation cost.

### Data processing

A total of 83 potentially related variables were preliminarily screened. After excluding three variables with > 20% of missing values, the remaining 80 variables were subjected to data pre-processing using the Classification And REgression Training (CARET) package in the R language. Thirteen variables showing a strong correlation (correlation coefficient > 0.9) with other independent variables were eliminated. The remaining 67 variables were then subjected to feature selection using the backward selection method, random forest sampling, and 10% cross-checking. Thereafter, the variables were ranked according to their importance. The 12 most important variables were retained.

Outliers were detected using the interquartile range (IQR), i.e., the difference between the upper and lower quartiles of the boxplot. The outliers were excluded and handled as missing values. Variables with > 20% missing values were deleted. The missing values of variables were replaced using multiple imputations.

### Model establishment

First, support vector machines (SVMs), decision trees C5.0, and extreme gradient boosting (XGBoost) were trained for three machine learning (ML) models using the following R packages: CARET, XGBoost, C50, e1071, and gbm. The hyperparameters were adjusted using a grid search. Thereafter, the CaretEnsemble^18^ was used to create ensemble models from these three models. Samples were randomly divided into training and test sets in a 7:3 ratio. All ML models were evaluated using 10 × cross-validation.

### Model validation and evaluation

The model performance was evaluated in the area under the receiver operating characteristic curve (AUC). The calibration performance of the model was evaluated using calibration curves. The confusion matrix was evaluated using accuracy, precision, specificity, and recall as parameters; the cut-off point was 0.5.

### Model interpretation

Model interpretation was implemented using variable importance, which was sorted using the function “varImp” within the CARET package in R. Moreover, local interpretable model-agnostic explanations (LIME) and iBreakdown algorithms provided individual interpretation^19,20^.

### Statistical analysis

Descriptive statistics were analysed conventionally using the CBCgrps package in R^21^. Normally distributed measurement data were expressed as *x* ± *s* and compared between groups using the two-independent-samples *t*-test. Meanwhile, non-normally distributed data were expressed as median (P25, P75) and compared using the Mann–Whitney U test. Enumeration data were expressed in terms of the rate and percentage and compared between the groups using the *χ*^2^ test. All statistical analyses were performed using R (software version 4.1.2). A *P*-value of 0.05 was considered significant.

## Results

### Study population and baseline characteristics

**Figure 1:**
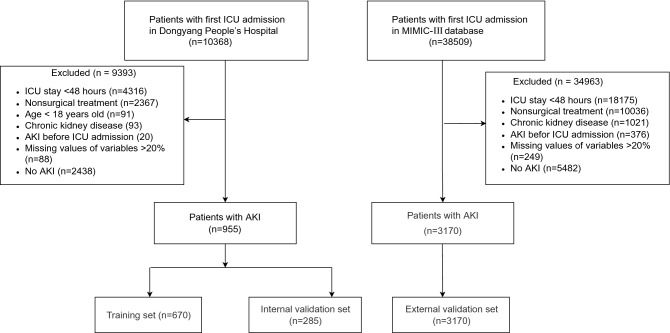
Flow chart of the study. ICU, intensive care unit; AKI, acute kidney injury.

Our study included a total of 955 patients complicated by AKI admitted to the ICU after surgery, including 376 patients with persistent AKI with an incidence of 39.4%; in the MIMIC III data set, the incidence was 45.1% (1429/3170 cases). The 955 patients with AKI were randomly divided into the training and internal verification sets at a ratio of 7:3. MIMIC III was used as the external verification. Figure 1 shows a flow chart of the study design. Table 1 presents a comparison of baseline data among the training, internal verification, and external verification sets.

**Table 1:** Comparison of feature distributions between the training, internal validation, and external validation sets.

Variables	Training (*n* = 670)	Internal validation (*n* = 285)	External validation (*n* = 3170)	*P*-value
Age (years), mean (SD)	63.5 ± 17	62.7 ± 16.6	64 ± 17.3	0.425
Male, *n* (%)	437 (65.2)	191 (67)	1882 (59.4)	0.002
SOFA (score), mean (SD)	7 ± 3.4	6.9 ± 3.5	5.1 ± 3	< 0.001
RRT, *n* (%)	52 (7.8)	17 (6)	100 (3.2)	< 0.001
Uo_6h (mL/kg/h), mean (SD)	0.2 ± 0.3	0.2 ± 0.3	0.3 ± 0.3	< 0.001
Uo_24h (mL/kg/h)	1.15 (0.73, 1.66)	1.25 (0.82, 1.85)	0.63 (0.43, 0.92)	< 0.001
**Comorbidities, *n* (%)**				
Hypertension	303 (45.2)	126 (44.2)	1589 (50.1)	0.018
Diabetes	87 (13)	38 (13.3)	766 (24.2)	< 0.001
**Biochemical indexes on ICU admission, mean (SD)**				
Creatinine (mmol/L)	107.1 ± 60.5	103.4 ± 58.5	83 ± 41.9	< 0.001
White blood cell (× 10^9^/L)	12.3 ± 6.4	12.8 ± 7	12.9 ± 6.1	0.072
pH	7.4 ± 0.1	7.4 ± 0.1	7.4 ± 0.1	0.542
Bicarbonate (mmol/L)	20.9 ± 3.8	20.7 ± 3.7	22.8 ± 3.7	< 0.001
Lactate (mmol/L)	3.4 ± 3.3	3.2 ± 3.1	2.7 ± 2	< 0.001
Urea (mmol/L)	9.2 ± 4.7	9.2 ± 4.5	18.3 ± 10.4	< 0.001
**Vital signs on first day ICU admission, mean (SD)**				
Maximum glucose (mmol/L)	11.7 ± 5.1	11.5 ± 4.6	190.7 ± 65.7	< 0.001
Mean systolic pressure (mmHg)	125.3 ± 17	125.1 ± 16.7	117.8 ± 14.8	< 0.001
Mean temperature (°C)	37.2 ± 0.7	37.2 ± 0.7	37 ± 0.6	< 0.001
Mean heart rate (bpm)	89.5 ± 18.6	90.4 ± 19.8	87.9 ± 14.8	0.004
**Outcome**				
Persistent AKI, *n* (%)	264 (39.4)	112 (39.3)	1429 (45.1)	0.008
ICU length of stay (days), median (IQR)	2.19 (0.73, 8.5)	1.86 (0.55, 7.82)	4.37 (2.97, 8.91)	< 0.001
Hosp. LOS (days), median (IQR)	22 (14, 33)	21 (14, 32)	11.08 (7.17, 17.88)	< 0.001
Hospital mortality, *n* (%)	114 (17)	57 (20)	348 (11)	< 0.001

SOFA, Sepsis-related Organ Failure Assessment; RRT, renal replacement therapy; Uo_6h, urine volume for 6 h on ICU admission; Uo_24h, urine volume for 24 h on ICU admission; ICU, intensive care unit; AKI, acute kidney injury; Hosp. LOS, length of hospital stay.

Table 2 shows a comparison of baseline data and clinical prognoses of patients in the persistent and transient AKI groups. There was no significant difference in age or sex. Comparison of SOFA and APACHE II scores showed that the severity of disease in the persistent AKI group was higher, and the clinical outcomes included time on ventilator, length of ICU stay, in-hospital mortality, and hospitalisation expenses. There were significant differences between the two groups. In addition, the use of contrast agents and diuretics was greater in the persistent AKI group. We also compared the baseline data and clinical outcomes of patients in the persistent and transient AKI groups in the MIMIC III data set, as shown in Supplementary Table S1.

**Table 2:** Comparisons of baseline characteristics and outcomes between patients with persistent and transient AKI.

Variables	Total (*n* = 955)	Transient AKI (*n* = 579)	Persistent AKI (*n* = 376)	*P*-value
Age (years), mean (SD)	63.3 ± 16.9	63.2 ± 17	63.5 ± 16.7	0.793
Male, *n* (%)	628 (65.8)	381 (65.8)	247 (65.7)	1
Smoking, *n* (%)	384 (40.2)	233 (40.2)	151 (40.2)	1
Alcohol drinking, *n* (%)	375 (39.3)	233 (40.2)	142 (37.8)	0.485
SOFA (score), mean (SD)	6.9 ± 3.4	6.4 ± 3.2	7.8 ± 3.6	< 0.001
APACHE II (score), mean (SD)	19.3 ± 7.2	18.6 ± 6.7	20.3 ± 7.8	< 0.001
Surgery time (hours), mean (SD)	3.1 ± 2.3	3 ± 2	3.2 ± 2.6	0.276
**Surgery, *n* (%)**				< 0.001
Abdominal	242 (25.3)	169 (29.2)	73 (19.4)	
Cerebral	172 (18)	84 (14.5)	88 (23.4)	
Orthopaedic	240 (25.1)	143 (24.7)	97 (25.8)	
Cardiothoracic	105 (11)	81 (14)	24 (6.4)	
Others	196 (20.5)	102 (17.6)	94 (25)	
Contrast agent use, *n* (%)	624 (65.3)	361 (62.3)	263 (69.9)	0.019
Antibiotic use, *n* (%)	43 (4.5)	14 (2.4)	29 (7.7)	< 0.001
Diuretic use, *n* (%)	778 (81.5)	445 (76.9)	333 (88.6)	< 0.001
**AKI stage**				< 0.001
1	588 (61.6)	424 (73.7)	161 (42.8)	
2	229 (24)	124 (21.4)	105 (27.9)	
3	138 (14.5)	28 (4.8)	110 (29.3)	
Uo_6h (mL), mean (SD)	324.8 ± 455.5	239.2 ± 400.5	456.7 ± 501.9	< 0.001
Uo_24h (mL), median (IQR)	1735 (1100, 2400)	1700 (1150, 2350)	1787.5 (1000, 2556.25)	0.792
**Comorbidities, *n* (%)**				
Hypertension	429 (44.9)	245 (42.3)	184 (48.9)	0.052
Diabetes	125 (13.1)	56 (9.7)	69 (18.4)	< 0.001
Myocardial infarction, *n* (%)	87 (9.1)	32 (5.5)	55 (14.6)	< 0.001
Chronic obstructive pulmonary disease	74 (7.7)	45 (7.8)	29 (7.7)	1
Solid tumour	110 (11.5)	79 (13.6)	31 (8.2)	0.014
Sepsis	550 (57.6)	337 (58.2)	213 (56.6)	0.683
**Biochemical indexes on ICU admission, mean (SD)**				
Creatinine (mmol/L)	106 ± 59.9	83.4 ± 32.4	140.8 ± 74.2	< 0.001
Urea (mmol/L)	9.2 ± 4.6	8.1 ± 3.5	10.9 ± 5.5	< 0.001
White blood cell (× 10^9^/L)	12.5 ± 6.6	12 ± 6	13.1 ± 7.4	0.016
Red blood cell (× 10^9^/L)	3.6 ± 0.8	3.6 ± 0.7	3.5 ± 0.9	0.024
Platelet count (× 10^9^/L)	151.4 ± 78.2	157.5 ± 79.8	142 ± 74.7	0.002
pH	7.4 ± 0.1	7.4 ± 0.1	7.3 ± 0.1	< 0.001
Bicarbonate (mmol/L)	20.9 ± 3.8	21.6 ± 3.1	19.7 ± 4.4	< 0.001
Lactate (mmol/L)	3.4 ± 3.2	2.7 ± 2.3	4.4 ± 4.1	< 0.001
Prothrombin time (s)	16.4 ± 6.2	15.6 ± 2.7	17.7 ± 9.2	< 0.001
Potassium (mmol/L)	4.2 ± 0.7	4.1 ± 0.6	4.3 ± 0.8	0.012
Sodium (mmol/L)	141.1 ± 4.9	140.7 ± 4.5	141.8 ± 5.5	0.002
**Vital signs on first day ICU admission, mean (SD)**				
Minimum glucose (mmol/L)	6.6 ± 1.8	6.5 ± 1.6	6.9 ± 2	0.006
Maximum glucose (mmol/l)	11.6 ± 4.9	10.8 ± 3.6	13 ± 6.3	< 0.001
Mean glucose (mmol/l)	8.9 ± 2.4	8.4 ± 2	9.6 ± 2.9	< 0.001
Minimum systolic pressure (mmHg)	92.9 ± 19.7	96.3 ± 19.1	87.8 ± 19.6	< 0.001
Maximum systolic pressure (mmHg)	179.2 ± 126.9	186.9 ± 151	167.4 ± 75	0.008
Mean systolic pressure (mmHg)	125.3 ± 16.9	127.8 ± 16.6	121.4 ± 16.8	< 0.001
Minimum diastolic pressure (mmHg)	49.9 ± 10.1	51.2 ± 9.8	47.9 ± 10.2	< 0.001
Maximum diastolic pressure (mmHg)	91.8 ± 43.5	90.4 ± 34.6	94 ± 54.4	0.244
Mean diastolic pressure (mmHg)	66.6 ± 9.8	67.1 ± 9.6	65.9 ± 10.1	0.088
Mean temperature (°C)	37.2 ± 0.7	37.2 ± 0.6	37.1 ± 0.8	< 0.001
Minimum temperature (°C)	36.1 ± 0.9	36.2 ± 0.8	35.9 ± 1.1	< 0.001
Maximum temperature (°C)	38.1 ± 0.8	38.1 ± 0.7	38 ± 0.9	0.552
Mean heart rate (bpm)	89.8 ± 19	86.5 ± 17	94.9 ± 20.7	< 0.001
Minimum heart rate (bpm)	69.6 ± 18.2	67.5 ± 15.9	72.8 ± 20.9	< 0.001
Maximum heart rate (bpm)	115.8 ± 27.2	111.7 ± 25.7	122 ± 28.2	< 0.001
**Clinical outcomes**				
ICU length of stay (days), median (IQR)	5.97 (3.67, 12.67)	5.23 (3.12, 11.63)	7.36 (4.51, 13.92)	< 0.001
Ventilation duration (days), median (IQR)	2.16 (0.7, 8.44)	1.65 (0.56, 7.74)	3.32 (0.81, 9.38)	0.002
Hosp. LOS (days), median (IQR)	22 (14, 33)	22 (14.5, 33)	22 (13, 33)	0.497
Cost (× 10^3^ yuan)	71.63 (46.73, 108.8)	64.18 (44.23, 94.55)	83.16 (52.1, 139.01)	< 0.001
Hospital mortality, *n* (%)	171 (17.9)	57 (9.8)	114 (30.3)	< 0.001

AKI, acute kidney injury; SOFA, Sepsis-related Organ Failure Assessment; APACHE, Acute Physiology and Chronic Health Evaluation; Uo_6h, urine volume for 6 h on ICU admission; Uo_24h, urine volume for 24 h on ICU admission; ICU, intensive care unit; Hosp. LOS, length of hospital stay.

### Model establishment and evaluation

**Figure 2:**
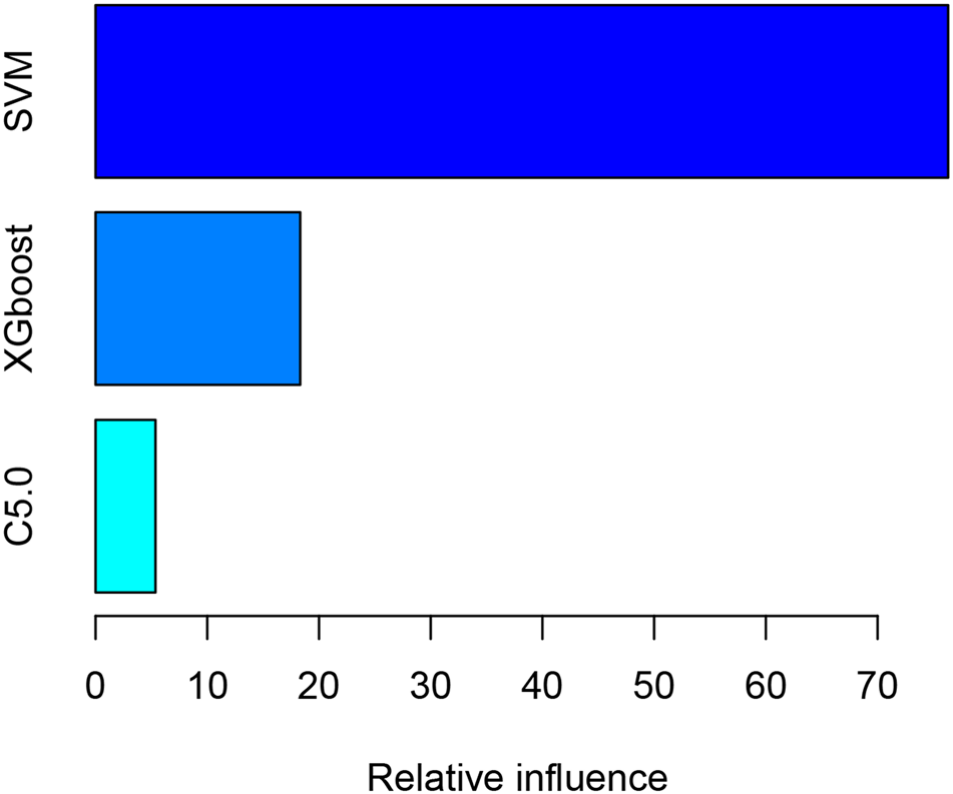
The relative influence of each machine-learning model in the stacked-ensemble model. SVM, support vector machine; XGBoost, extreme gradient boosting.

**Figure 3:**
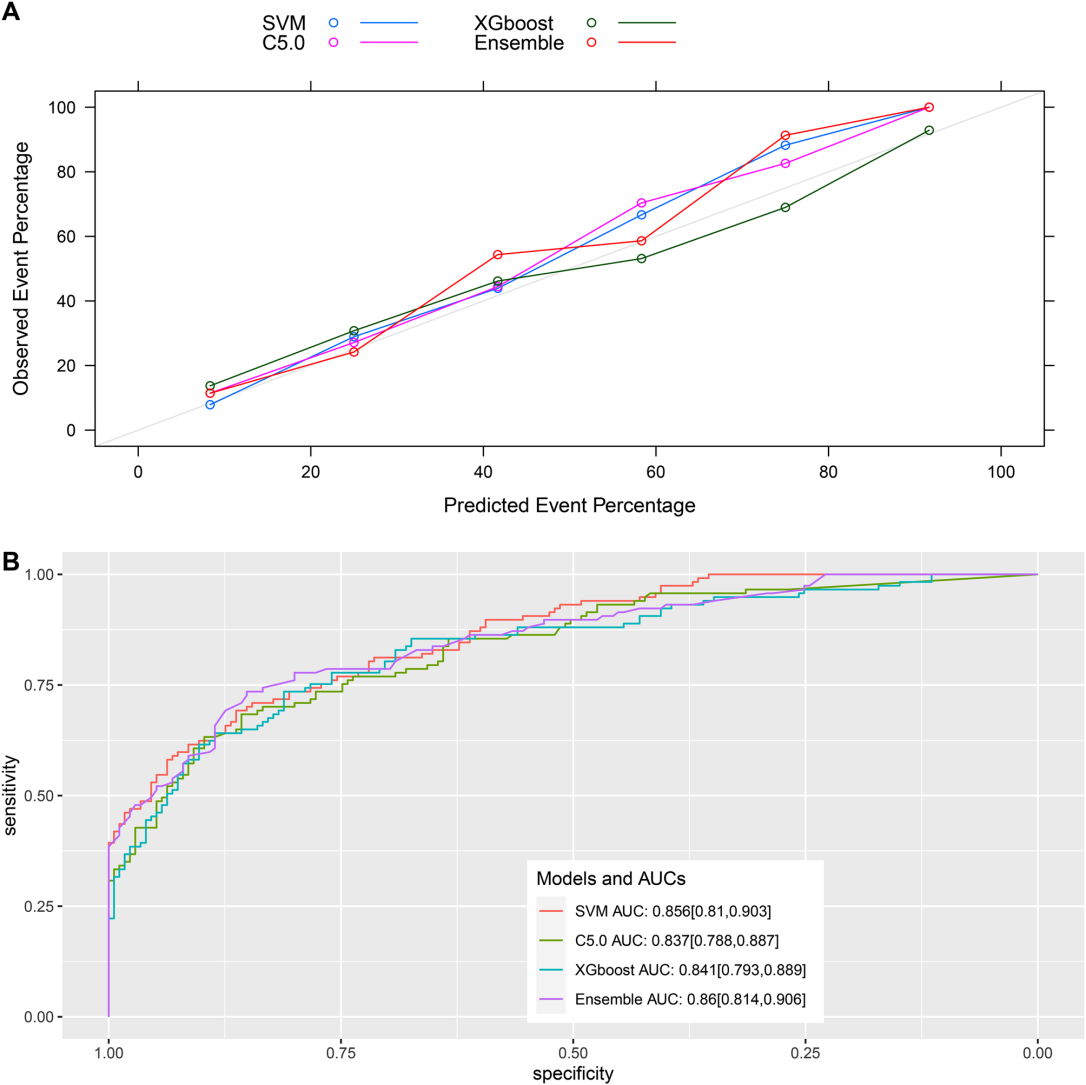
Evaluation of model performance in the internal validation dataset. (**A**) The calibration plot shows the consistency between observed and predicted risks for persistent acute kidney injury. (**B**) Discrimination of the machine-learning models in the internal validation dataset. SVM, support vector machine; XGBoost, extreme gradient boosting; AUC, area under the curve. The number in parentheses indicates the 95% confidence interval.

We used ML algorithms C5.0, SVM, and XGBoost to establish models. Supplementary figure S1−3 illustrate the hyperparameter tuning. We created an integrated model using these three models. The relative influence of each ML model in the integrated model is shown in Fig. 2, which shows that SVM and XGBoost greatly influenced the integrated model. Figure 3 presents the comparison of the prediction performance of machine learning C5.0, SVM, XGBoost, and integrated models in the internal verification set. The model calibration chart shows that all models had good predictive performance, with the integrated model showing the highest AUC (0.86, 95% CI: 0.814–0.906), and the SVM showing the second highest AUC (0.856, 95% CI: 0.812–0.901). The ROC comparison and model calibration chart in the external verification set are shown in Supplementary figure S4. The AUC value of the SVM was 0.693 (95% CI: 0.676–0.711). Supplementary Table S2 lists other evaluation indexes of prediction performance of the ML models.

### Model interpretation

**Figure 4:**
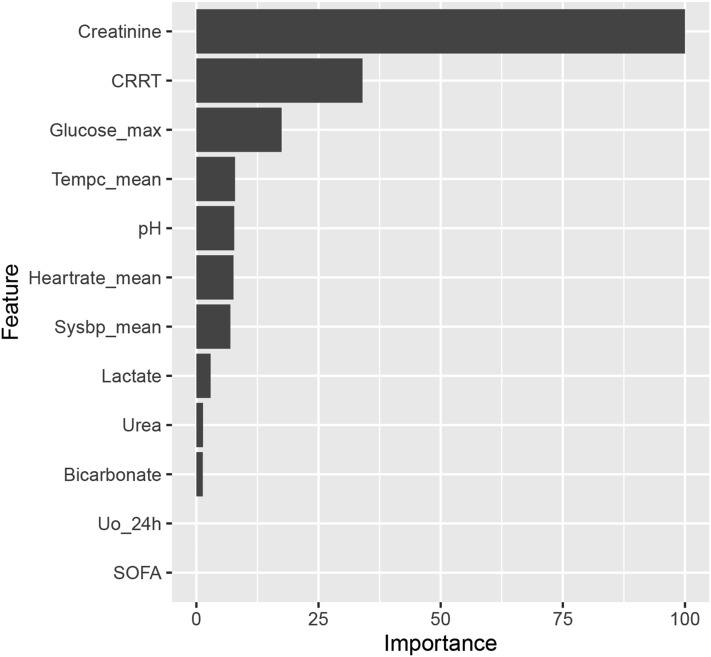
Variable-importance ranking in the gradient-boosting machine. RRT, renal replacement therapy; SOFA, Sepsis-related Organ Failure Assessment; Uo_24h, urine volume for 24 h on ICU admission.

**Figure 5:**
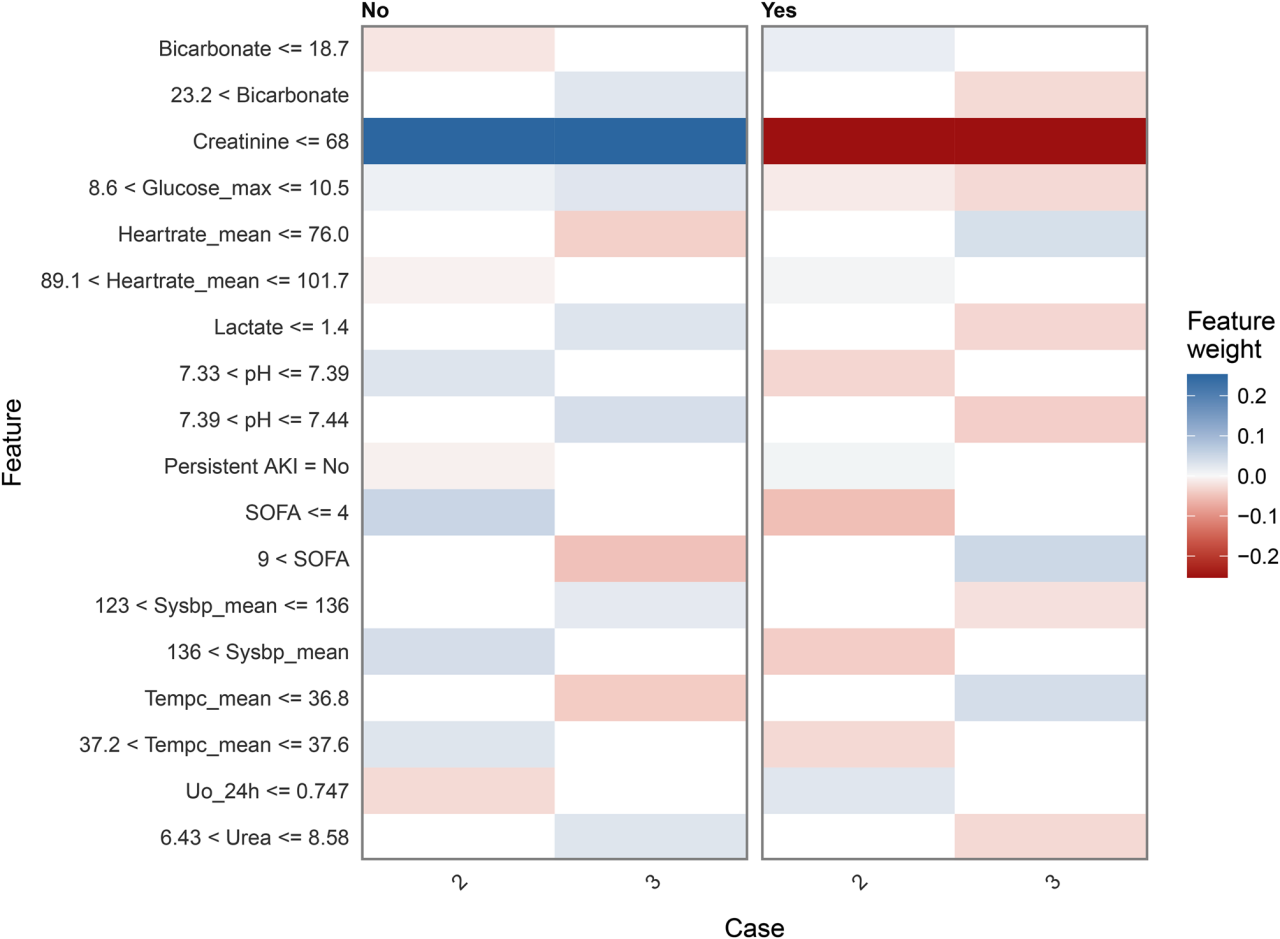
Heatmap plot showing the contribution of each variable to the classification of the sample patients. The relative contribution of each variable was calculated using the LIME algorithm. Data of patients 2 and 3 are shown as examples. The red colour indicates that the relevant variable contradicts a given label, while the blue colour indicates support. AKI, acute kidney injury; SOFA, Sepsis-related Organ Failure Assessment; Uo_24h, Urine volume for 24 h on intensive care unit admission; LIME, Local Interpretable Model-Agnostic Explanations.

**Figure 6:**
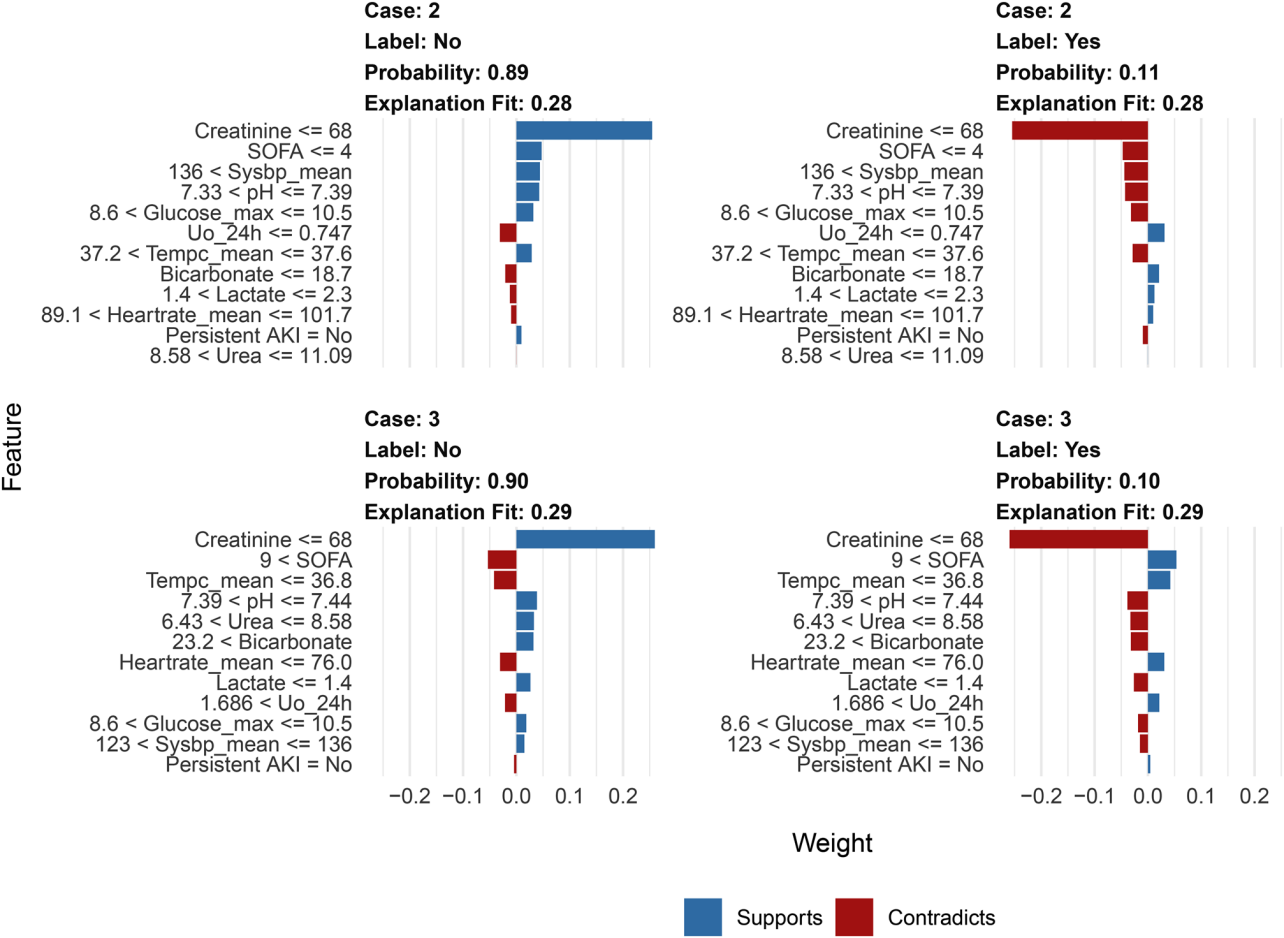
The LIME feature plot shows the contribution of each variable to the classification of the sample patients. The red colour indicates that the relevant variable contradicts a given label, while the blue colour indicates support. AKI, acute kidney injury; SOFA, Sepsis-related Organ Failure Assessment; Uo_24h, Urine volume for 24 h on intensive care unit admission; LIME, Local Interpretable Model-Agnostic Explanations.

Figure 4 and Supplementary Figures S5 and S6 show the order of importance of variables in the models. The Cr level, RRT, and lac level on ICU admission exhibited the greatest influence on the models. LIME provided interpretation for individual patients, and we estimated the contribution of the variables of two patients (Patients 2 and 3), as shown in Figs. 5 and 6. We used the iBreakdown method (Supplementary Figure S7) to estimate the contribution of each variable to the probability of persistent AKI for Patient 1, which showed that the initial Cr = 56 was closely correlated with a reduced risk of persistent AKI.

## Discussion

### Principal results

Our research utilised vital signs and related information in the 24 h after admission to the ICU, which could distinguish between persistent and transient AKI in advance, based on SVM or ML integration algorithms. The AUCs in the internal and external verification sets were approximately 0.86 and 0.69, respectively, showing that the model detection performance was good. The prediction effect of the integrated ML model was not as good as that of the SVM in the external verification sets, which might be related to the model we chose. We combined only three common ML algorithms: C5.0, SVM, and XGBoost. In a previous study, Rahman et al. integrated ML elastic network regression, random forest, and XGBoost to predict the early recurrence of oesophageal cancer after surgery, and the best AUC of the integrated ML algorithm was 0.805, whereas the AUC of XGBoost was 0.804, with a slight difference between them^22^. Similarly, super learner, an algorithm specifically integrating ML, has great potential in improving prediction quality in applied health science^23^. It contains a large number of ML algorithms, and the number of applications is increasing. Therefore, our models require further improvement. By integrating more ML algorithms, the performance of the model may be further improved in the future.

### Comparison with prior work

Previous research has shown that the incidence of persistent AKI varies with the severity of the disease. For example, in a large retrospective cohort study, involving over 54,000 patients with AKI from 2012 to 2019, the incidence of persistent AKI was 42%^24^. In a prospective study among ICU patients, the proportion of patients with persistent AKI among ICU patients at six hospitals was as high as 61.8% (175/283)^25^. Our research showed that the incidence of persistent AKI in ICU patients with AKI after surgery was 39.4–45.1% and was relatively low among ICU patients; this was because AKI was correlated with reversible factors such as hypovolaemia and ischaemia, which were common in postoperative patients. Early identification of these patients can help to identify the phenotypes that require different treatment schemes, thereby reducing excessive intervention treatment.

In the past few years, there has been an increasing number of prediction models for AKI based on ML, but few studies have focused on ICU patients. A study by Ding et al., which used free databases to screen important variables that affect persistent AKI based on ML algorithms, and the nomogram produced showed good prediction performance^26^. Another study by Luo et al. used five ML algorithms to identify persistent AKI in ICU patients with sepsis at an early stage, and the AUC of the model with the optimal algorithm reached 0.76. However, most of these studies only used creatinine as the standard for AKI diagnosis, and generally lacked external verification^27^. Our research included creatinine and urine volume data, and used MIMIC data for verification, which made the model suitable for more general condition.

### Limitations

First, this study was retrospective in nature, and urine volume was not recorded every hour. Therefore, when we calculated urine volume for 6 h, the data were inaccurate, and for patients transferred to other departments or discharged from the hospital within 48 h following admission to the ICU, urine volume data was not collected. However, our data are more consistent with actual practice than when only a creatinine standard is used to diagnose AKI. Second, there is a black box effect in ML. We explained the models by three methods, namely rank of variable importance in the models, LIME, and iBreakdown, which allows individualised application to patient. Finally, 12 variables were included in the models, including the maximum and average vital signs on first day after ICU admission. The method of calculation was somewhat complicated. It is better to automatically identify persistent AKI by adding data to the current electronic medical record system.

## Conclusions

Critically ill patients with AKI after surgery displayed a high incidence of persistent AKI and poor prognoses. We could predict the occurrence of persistent AKI in advance using the machine learning models and the integrated model; external verification by the MIMIC III database, and the model calibration was good. In the future, the electronic medical record system can be integrated to automatically identify persistent AKI to facilitate individualised treatment and improve patient outcomes.

## Supplementary Information

Supplementary Information.

## Data Availability

The datasets of Dongyang People’s Hospital are available from the corresponding author on reasonable request. The MIMIC III dataset is a freely accessible online critical care database (https://mimic.physionet.org/).
