# Supplementary material for: Prediction of persistent acute kidney injury in postoperative intensive care unit patients using integrated machine learning: a retrospective cohort study
Source: Sci Rep. 2022 Oct 12;12:17134. doi: 10.1038/s41598-022-21428-5 (PMC9556643; doi:10.1038/s41598-022-21428-5)
Supplement: Supplementary file 1 — Supplementary Information. [file 41598_2022_21428_MOESM1_ESM.pdf]

Supplementary Information

Prediction of persistent acute kidney injury in postoperative intensive care unit patients using integrated machine learning: A retrospective cohort study

Xuandong Jiang*[,1], Yongxia Hu[1], Shan Guo[1], Chaojian Du[1], Xuping Cheng[1]

[1] Intensive Care Unit, Affiliated Dongyang Hospital of Wenzhou Medical University, Dongyang, Zhejiang, P.R. China

Corresponding author: Xuandong Jiang, MD, Intensive Care Unit, Affiliated Dongyang Hospital of Wenzhou Medical University, Dongyang, Zhejiang, P.R. China, No. 60 Wuning West Road, Jinhua, China.

Email: lxqjiang@hotmail.com.

Table S1 Baseline characteristics and clinical outcomes between persistent AKI and transient AKI groups in the MIMIC Ⅲ cohort

Variables	Transient AKI (n = 1741)	Persistent AKI (n = 1429)	P-value
Age (years), mean (SD)	1022 (58.7)	860 (60.2)	0.419
Male, n (%)	61.7 ± 18.5	66.9 ± 15.4	< 0.001
SOFA (score), mean (SD)	4.4 ± 2.6	6 ± 3.1	0.32
Height (cm), mean (SD)	169.6 ± 10.6	169.3 ± 11.3	0.32
Weight (kg), mean (SD)	79.7 ± 18.6	84.8 ± 23.8	< 0.001
AKI stage, n (%)			< 0.001
1	868 (49.9)	368 (25.8)	
2	779 (44.7)	753 (52.7)	
3	94 (5.4)	308 (21.6)	
admission_type, n (%)			< 0.001
ELECTIVE	375 (21.5)	393 (27.5)	
EMERGENCY	1339 (76.9)	1004 (70.3)	
URGENT	27 (1.6)	32 (2.2)	
Comorbidities, n (%)			
Coronary	531 (30.5)	576 (40.3)	< 0.001
Hypertension	868 (49.9)	721 (50.5)	0.764
Diabetes	353 (20.3)	413 (28.9)	< 0.001
Biochemical indexes on ICU admission			
White blood cell (× 109/L), mean (SD)	13 ± 6.1	12.8 ± 6.2	0.573
Urea (mmol/L), mean (SD)	15 (12, 19)	18 (13, 23)	< 0.001
pH, mean (SD)	7.4 ± 0.1	7.4 ± 0.1	0.006
Lactate (mmol/L), median (IQR)	2 (1.3, 3.1)	2.2 (1.5, 3.4)	< 0.001
Bicarbonate (mmol/L), mean (SD)	22.9 ± 3.6	22.8 ± 3.8	0.31
Vital signs on first day ICU admission			
Minimum glucose (mmol/l), mean (SD)	101.9 ± 28.7	95.9 ± 29.9	< 0.001
Maximum glucose (mmol/l), median (IQR)	173 (147, 205)	183 (157, 218)	< 0.001
Mean glucose (mmol/l), median (IQR)	131.83 (119, 151.75)	131.83 (119.81, 152.89)	0.473
Minimum heart rate (bpm), mean (SD)	72.7 ± 14.5	71.5 ± 14.2	0.016
Maximum heart rate (bpm), mean (SD)	107.2 ± 20.3	106.6 ± 20.5	0.426
Mean heart rate (bpm), mean (SD)	88.3 ± 14.9	87.5 ± 14.6	0.121
Minimum temperature (°C),	36 ± 0.9	35.8 ± 1	< 0.001

mean (SD)			
Maximum temperature (°C), mean (SD)	37.8 ± 0.8	37.7 ± 0.8	< 0.001
Mean temperature (°C), mean (SD)	37 ± 0.6	36.9 ± 0.7	< 0.001
Outcome			
ICU length of stay (days), median (IQR)	4.19 (2.95, 7.89)	4.95 (2.98, 10.44)	0.002
Hosp. LOS (days) ,median (IQR)	10.58 (7.12, 17)	11.96 (7.21, 19.67)	< 0.001
Hospital mortality, n (%)	130 (7.5)	218 (15.3)	< 0.001

AKI, acute kidney injury; MIMIC, Medical Information Mart for Intensive Care; SOFA, Sepsis-related Organ Failure Assessment; ICU, intensive care unit; Hosp. LOS, length of hospital stay.

Table S2. Comparison of the additional evaluation metrics of four machine learning models in internal and external validation

Models for Predicting Persistent AKI in Internal Validation				
	SVM	C5.0	XGBoost	Ensemble
Accuracy	0.81	0.78	0.79	0.81
Precision	0.75	0.71	0.72	0.75
Recall	0.79	0.74	0.77	0.79
Specificity	0.83	0.80	0.80	0.83
Models for Predicting Persistent AKI in External Validation				
	SVM	C5.0	XGBoost	Ensemble
Accuracy	0.65	0.62	0.63	0.64
Precision	0.64	0.62	0.64	0.65
Recall	0.59	0.53	0.53	0.51
Specificity	0.70	0.71	0.72	0.75

AKI, acute kidney injury; SVM, support vector machine; XGBoost, extreme gradient boosting.

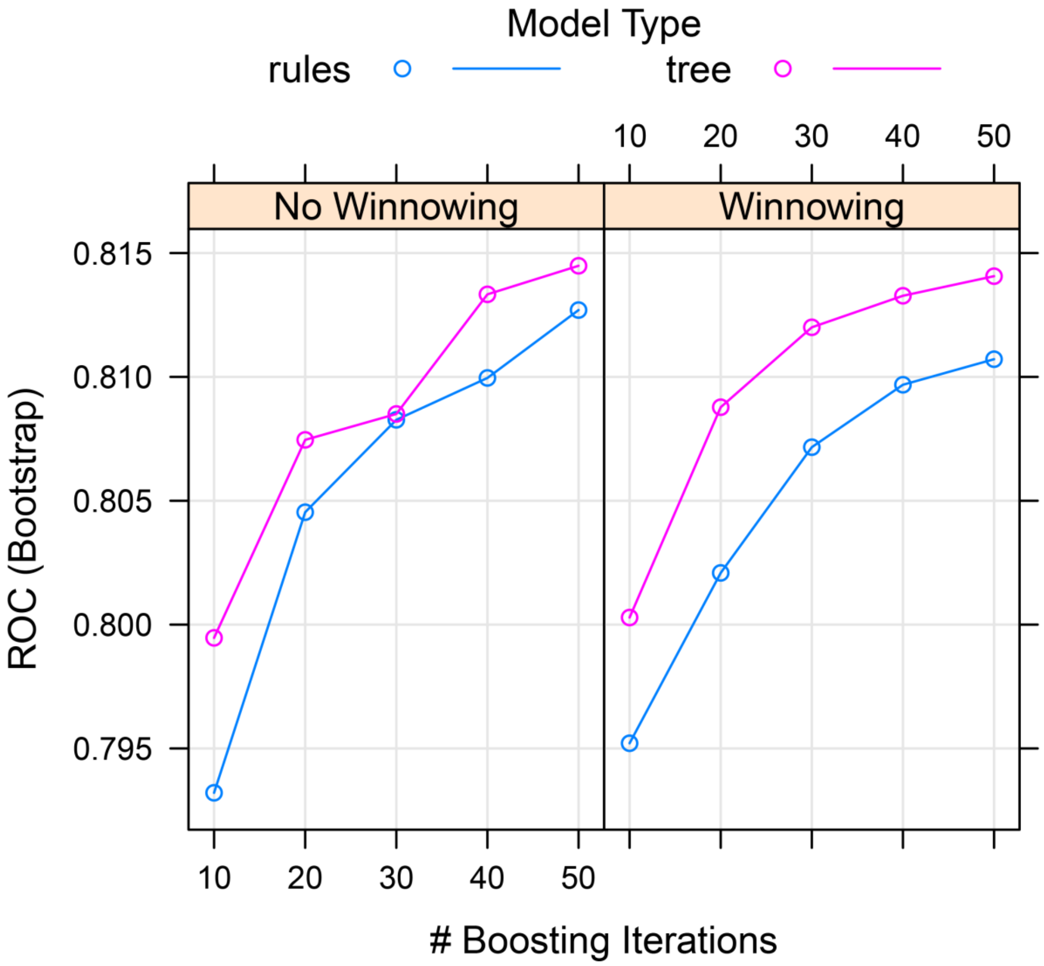

Figure S1. Hyperparameter tuning for the C5.0 machine-learning algorithm. Abbreviations: ROC, receiver operating characteristic.

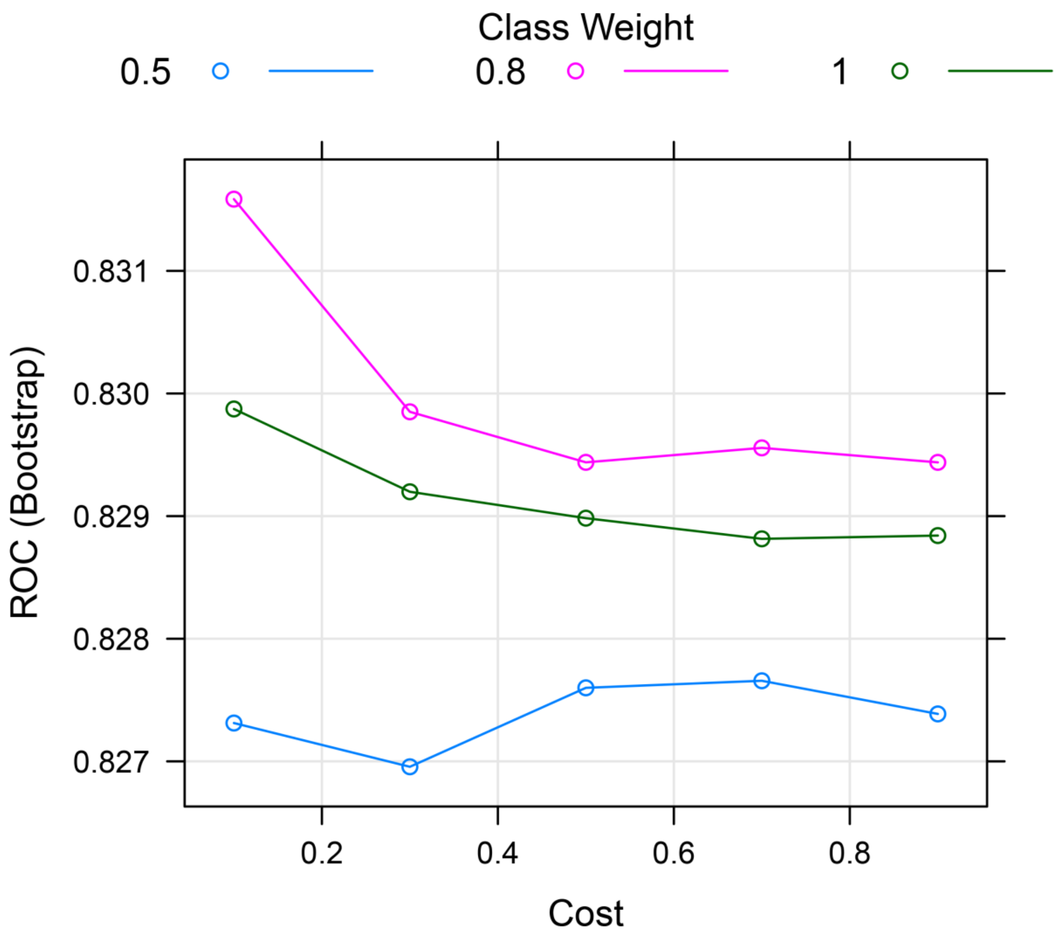

Figure S2. Hyperparameter tuning for the support vector machine machine-learning algorithm. Abbreviations: ROC, receiver operating characteristic.

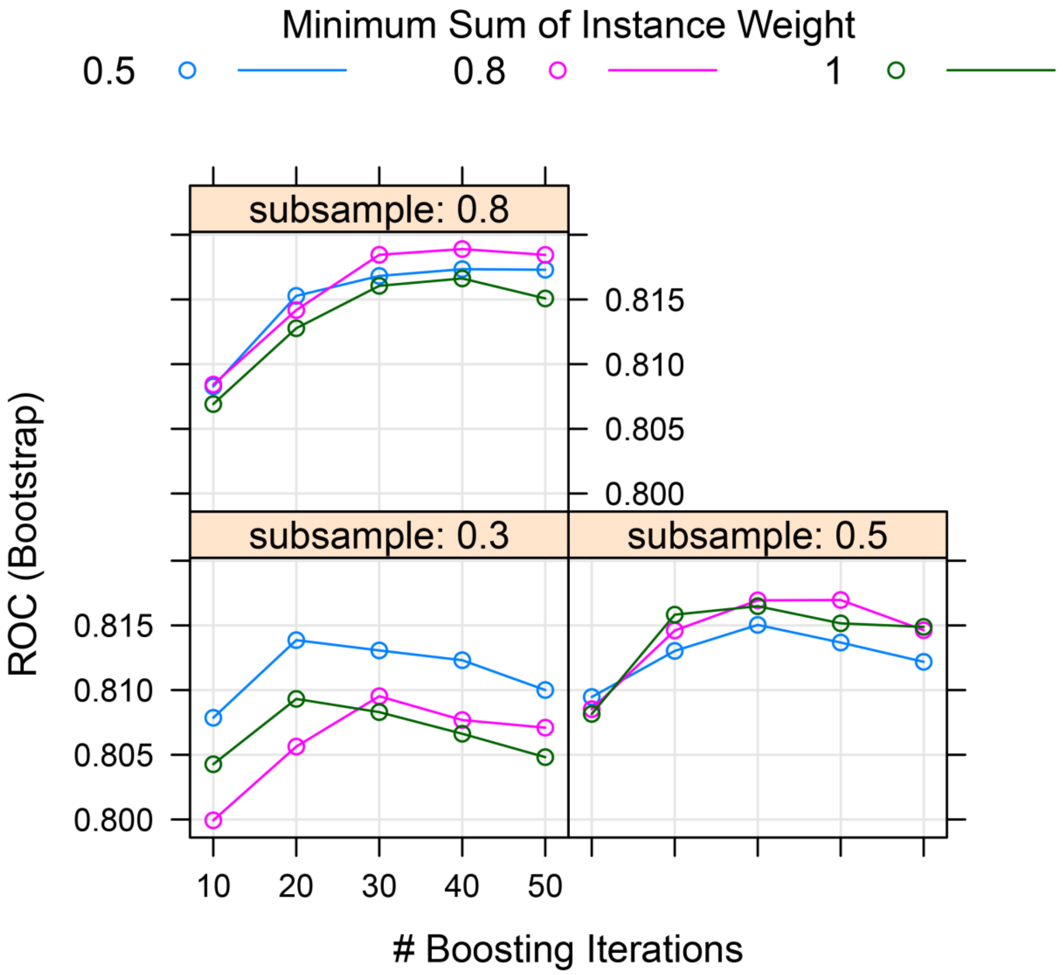

Figure S3. Hyperparameter tuning for the extreme gradient boosting machine-learning algorithm. Abbreviations: ROC, receiver operating characteristic.

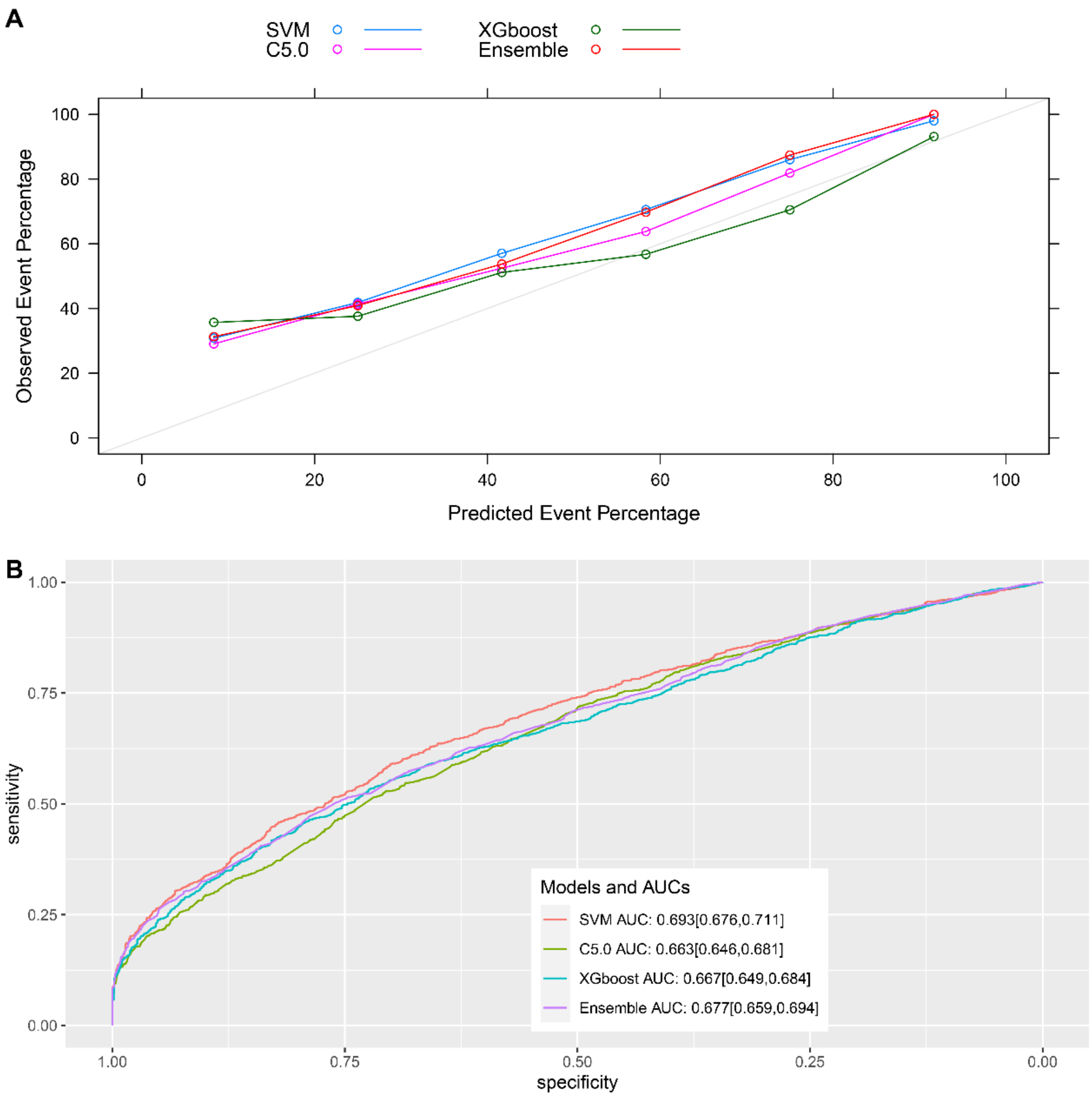

Figure S4. Evaluation of model performance in the external validation dataset. A) The calibration plot shows the consistency between observed and predicted risks for persistent acute kidney injury. B) Discrimination of the machine-learning models in the external validation dataset. SVM, support vector machine; XGBoost, extreme gradient boosting; AUC, area under the curve. The number in parentheses indicates the 95% confidence interval.

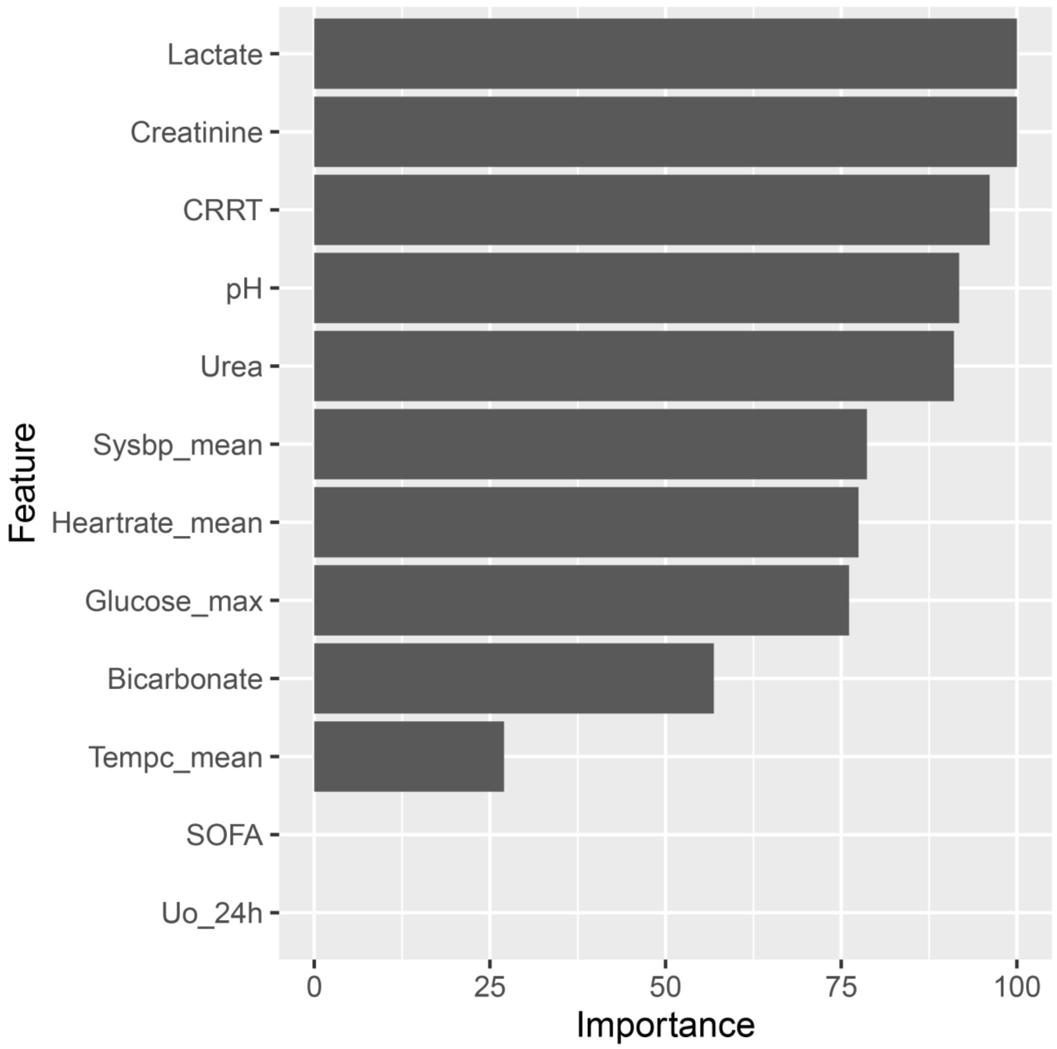

Figure S5. Variable-importance ranking in the C5.0. RRT, renal replacement therapy; SOFA, Sepsis-related Organ Failure Assessment; Uo_24h, urine volume for 24 hours on ICU admission.

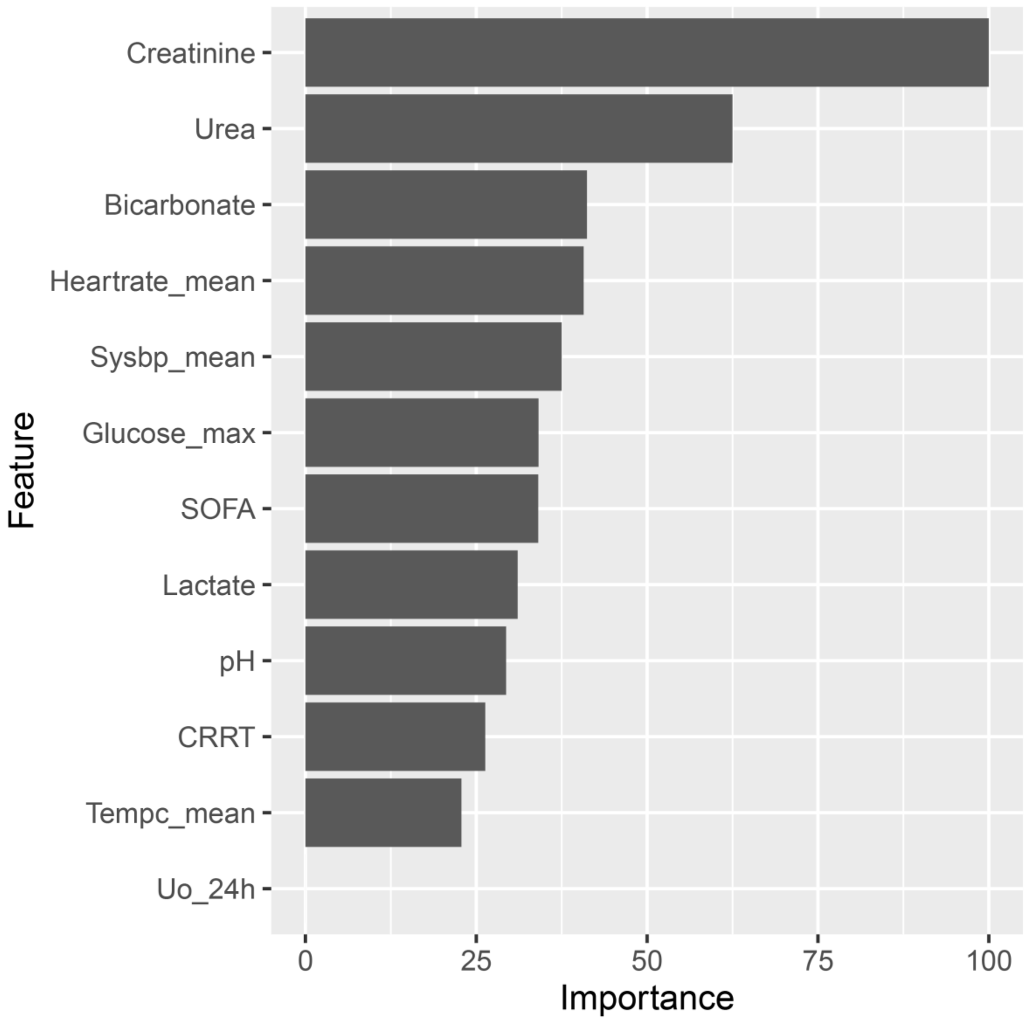

Figure S6. Variable-importance ranking in the support vector machine. RRT, renal replacement therapy; SOFA, Sepsis-related Organ Failure Assessment; Uo_24h, urine volume for 24 hours on ICU admission.

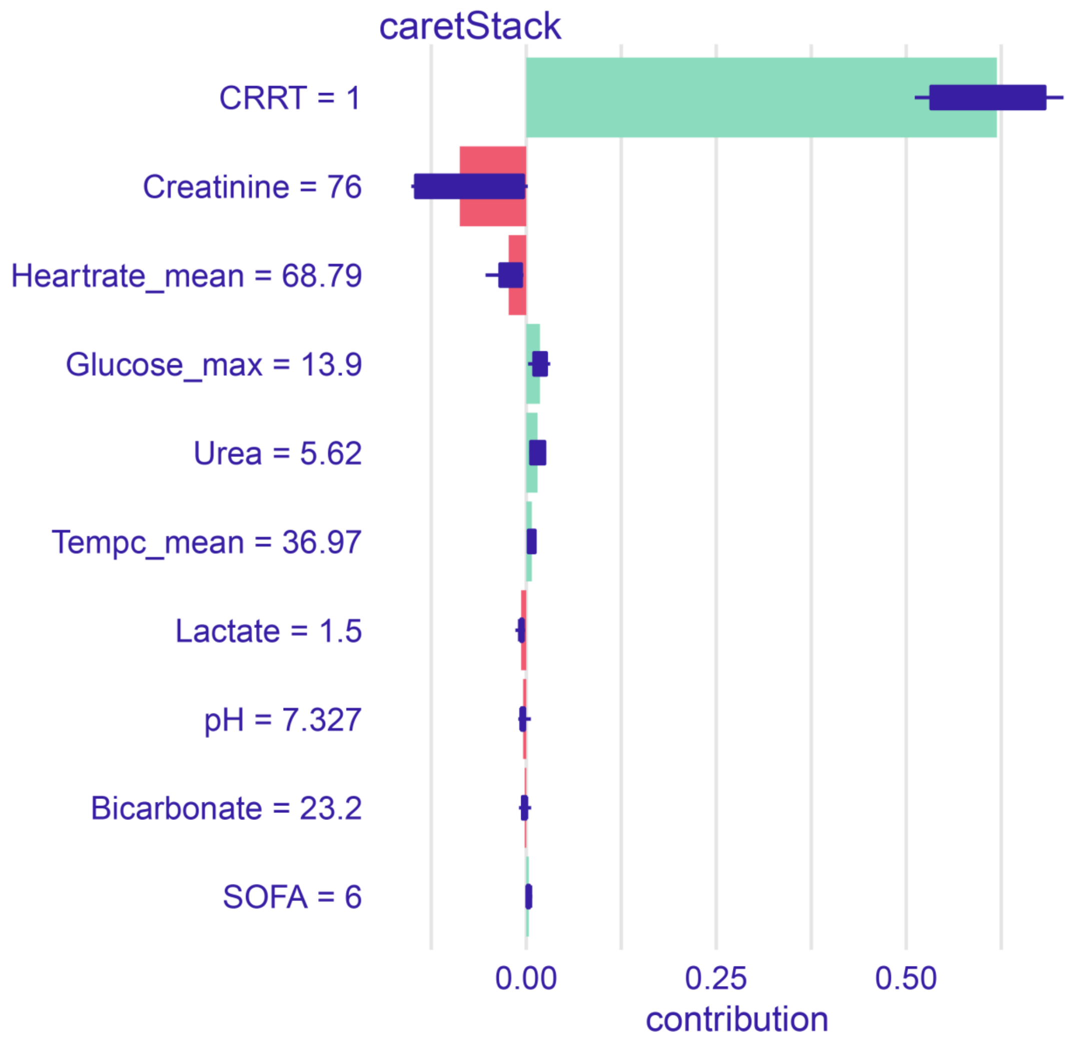

Figure S7. Model interpretation using the iBreakdown algorithm with uncertainty indicated by a box plot. The horizontal axis reflects the probability scale. RRT: renal replacement therapy; SOFA: Sepsis-related Organ Failure Assessment; Uo_24h: Urine volume for 24 hours on intensive care unit admission.
